# Uncovering plant root traits and mechanisms that enable penetration, exploration, and exploitation of soil parent materials: a systematic review

**DOI:** 10.1007/s11104-025-07916-3

**Published:** 2025-10-06

**Authors:** Paul Chaibva, Christopher S. McCloskey, Tom Sizmur, Lucy M. Greenfield, Sharif Ahmed, Daniel L. Evans

**Affiliations:** 1 Centre for Soil, Agrifood and Biosciences, Cranfield University, Cranfield, Bedfordshire UK; 2 Department of Geography and Environmental Science, University of Reading, Reading, Berkshire UK; 3 Department of Botany, University of Otago, Dunedin, New Zealand; 4 Diamond Light Source, Rutherford Appleton Laboratory, Didcot, Oxfordshire UK; 5 Department of Agronomy and Horticulture, Midlands State University, Gweru, Zimbabwe

**Keywords:** Root traits, Root mechanisms, Soil parent materials, Root penetration, Mechanical impedance, Soil compaction

## Abstract

**Background and aims:**

Certain plant species, including some trees, have been observed growing not only in soil but also in soil parent materials. However, the root traits and mechanisms enabling these species to penetrate soil parent materials are not yet thoroughly understood. This systematic review aims to identify and discuss the root traits and mechanisms that allow plant roots to grow into soil parent materials. It will also draw insights from the characteristics and mechanisms that plants employ to overcome the challenges posed by compacted soils.

**Methods:**

We adhered to the 'Preferred Reporting Items for Systematic Reviews and Meta-Analyses' (PRISMA) guidelines for our methodology.

**Results:**

We identified increased root radial pressure, investment in root biomass, fine root development, root trematotropism, mycorrhizal associations, root hairs, and root exudates as key traits aiding plants in soil penetration. The mentioned root traits and mechanisms have also been shown to help plants overcome compacted soil, except for mycorrhizal associations.

**Conclusion:**

The key root traits and mechanisms identified in this review lay the groundwork for a deeper understanding of root-soil parent material interactions and plant adaptations in changing physical environments. This enhances our ability to select the next generation of robust and resilient crops capable of thriving in complex root-soil parent material interactions. Future research on root-parent material interactions in food crops holds promise for improving our understanding of how crops can grow beyond traditional soil limitations (such as soil depth).

**Supplementary Information:**

The online version contains supplementary material available at 10.1007/s11104-025-07916-3.

## Introduction

Soil erosion poses one of the most severe threats to global food production due to its detrimental impacts on productivity and biogeochemical cycles (Alewell et al. 2020). Soil erosion is a natural process, but erosion rates have accelerated over the past century due to intensive cultivation practices, which destroy soil structure and increase the susceptibility of soil to erosion (Kanianska et al. 2024; Rhodes 2014). To meet the food demands of the projected global population of 10 billion by 2050, the agriculture sector must develop crops capable of yielding higher outputs in degraded soils (Springmann et al. 2018). A high rate of soil erosion, outpacing natural soil formation rates, results in thinner soils, exposing the underlying soil parent material. This also increases the risk of flooding as the soil parent material provides a semi-permeable or impermeable layer that hinders water infiltration (Rubinato et al. 2019). Flooding stress negatively affects crop production by suffocating roots and can cause yield losses up to 100% (Kaur et al. 2020). Soil erosion contributes to soil thinning, but leptosols, which are naturally shallow to about 30 cm due to dense, slow-weathering parent material beneath, must also be acknowledged (Spaargaren 2001). Terminology for materials beneath subsoils varies by discipline; in this review, ‘soil parent materials’ refer to the geological materials that underlie soil, including saprolite/saprock (C and R horizons), partly weathered and unweathered bedrock. These soil parent materials differ in their degree of weathering, which is correlated to their degree of mechanical impedance and resistance to penetration (bulk densities and penetrative resistance ranging 1.65–2.34 g cm^–3^, 2.0–6.0 MPa) (Graham et al. 1997; Stephan et al. 2015).

Mechanical impedance plays a significant role in restricting root elongation, which, in turn, promotes radial growth of an individual root (Feng et al. 2020). This condition poses a serious threat to agricultural food production and global food security, particularly in regions with shallow soils that hinder the deep rooting of plants (Tracy et al. 2011). By limiting root penetration to shallow topsoil, mechanical impedance often leads to poor spatial distribution of root systems within the soil matrix, negatively impacting crop yield (Tracy et al. 2011). The depth of the soil determines the volume of resources available to plants for fulfilling their nutritional, water, and anchorage requirements. For maximum growth, crops need their effective rooting depth (the part of the soil where most plant roots are present) to provide sufficient water and nutrients (Irmak and Rudnick 2014). Shallow soil depth can restrict crop yields by limiting access to necessary water and nutrients (Gardner et al. 1999). Furthermore, even when water and nutrients are available, mechanical impedance to root growth in shallow soils can hinder plant development (McConnaughay and Bazzaz 1991; Passioura 2002). Therefore, crops that grow in shallow soils may need to penetrate and grow through the soil parent material to maximise their yield. In such a case, the soil parent material would serve as a substantial source of nutrients (Gray and Murphy 2002). To enhance food security in areas with shallow soils, we need robust crops that can overcome significant mechanical impedance beneath, access vital nutrients and water, and anchor themselves in the underlying soil parent material.

While it has been shown that mechanical impedance significantly influences the roots of crops, it does not seem to have the same effect on the roots of trees and certain grass species. The ability of many tree species to grow through soil and into the soil parent material is well documented. According to Williams and Vepraskas (1994), tree roots can penetrate chemically weathered yet consolidated soil parent material, despite its low porosity and high bulk density (1.65–2.34 g cm^–3^) (Graham et al. 1997). In addition to tree roots, some grass species can also penetrate saprolite. Doerge and Smith (2008) noted the successful growth of Bahia grass (*Paspalum notatum* Flugge) and vetiver grass (*Vetiver zizanioides* L.) on bare saprolite. There is considerable evidence indicating that plants require specific adaptations to thrive in such environments (Schwinning 2010; Zwieniecki and Newton 1995). While tree roots have been shown to extend through the soil into the underlying saprolitic layer, little is known about the precise root traits and mechanisms that enable this growth. In their efforts to understand the key traits of *Hakea* species of the Proteaceae family that enable them to thrive in shallow soils, Poot and Lambers (2008) compared *Hakea* species that are typically found in shallow soils with their counterparts usually found in deeper soils. They observed that the species have a specialised root architecture that allows them to explore, increasing their chances of finding cracks in the underlying rock. Their research is one of the few attempts to explain the root traits and mechanisms that give some species the ability to penetrate and grow into soil parent material. Indeed, the adaptive traits and mechanisms facilitating root penetration into soil parent materials are not well documented in the literature and have not been thoroughly reviewed. This highlights a significant research gap that this systematic review aims to address. In this review, the term ‘root trait’ refers to the physical characteristics of plant roots, while root mechanism refers to the functional processes and strategies of the roots. Additionally, in this review, although we recognise the influence of abiotic factors (water/moisture, temperature, aeration, soil texture, nutrient availability, salinity, pH, etc.) on root penetration and development under mechanical impedance, we did not discuss these factors separately; instead, we focused on different levels of mechanical impedance under consistent abiotic conditions. Furthermore, various lithologies provide different mechanical resistances to roots, but there is limited literature available to discuss this.

This systematic review utilises literature examining root growth and development in compacted soils to propose traits and mechanisms that may be relevant for penetration, exploration, and resource acquisition in soil parent materials. There is a lack of research specifically assessing root traits or mechanisms enabling plant roots to grow and exploit soil parent materials. Although notable chemical, physical, and structural differences exist between compacted soils and soil parent materials, their bulk densities and penetrative resistance slightly overlap [(compacted soils (0.86–1.91 g cm^–3^, 0.5–2.5 MPa) (Olowolafe 2002; Grzesiak et al. 2013) and soil parent materials (1.65–2.34 g cm^–3^, 2.0–6 MPa) (Graham et al. 1997; Stephan et al. 2015)]. Therefore, the similarities between root traits and mechanisms that enable roots to grow and penetrate both compacted soils and soil parent materials are worth exploring. This systematic review will emphasise the need for resilient crop varieties that can grow sustainably on shallow soils while maintaining high yields. Developing crops capable of rooting in soil parent materials will facilitate the optimal use of shallow soils by reclaiming degraded land and transforming it into productive croplands.

## Systematic review of literature

### Methodology

**Fig. 1:**
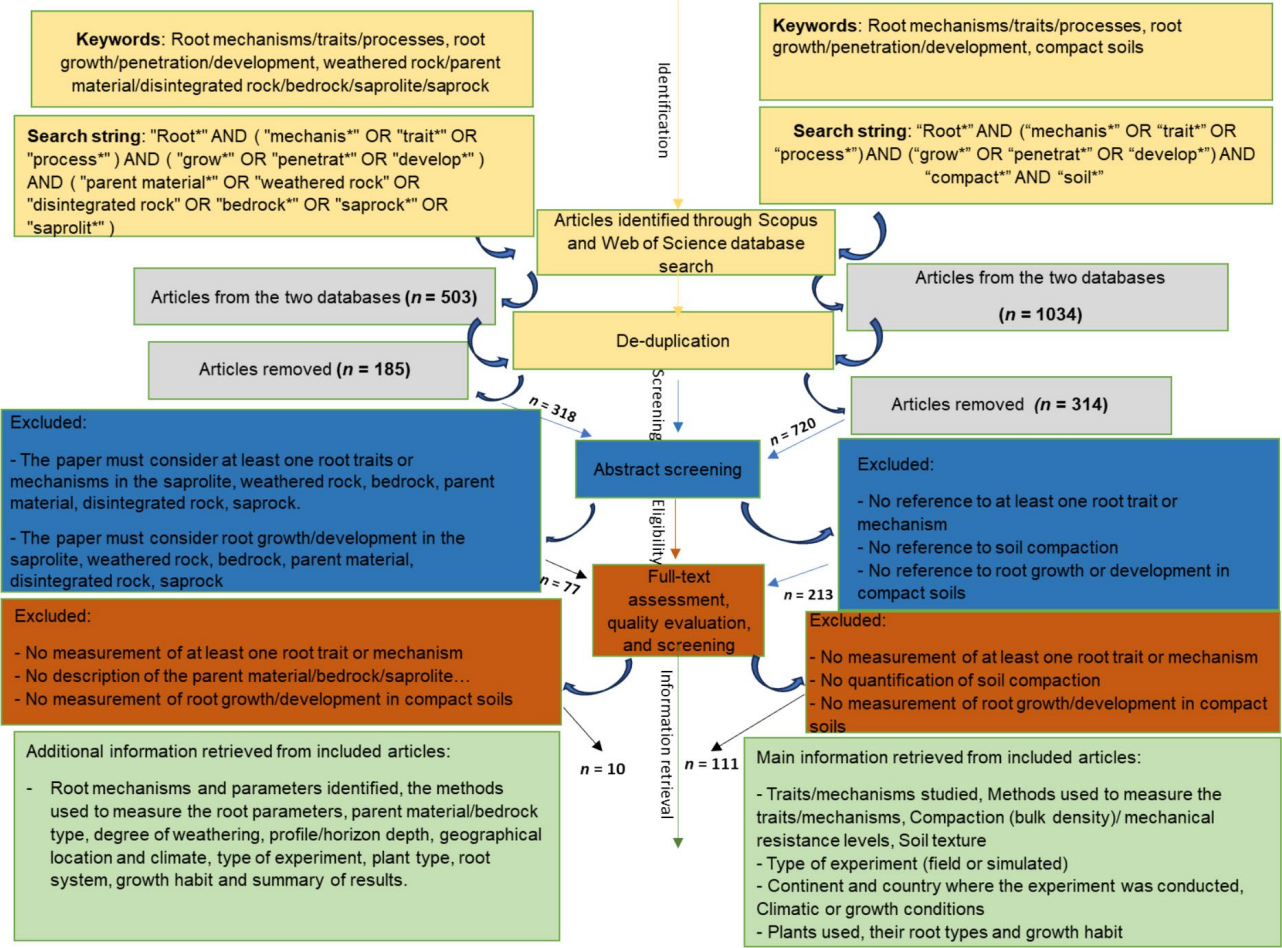
PRISMA flowchart summarising the systematic review process for identifying and processing articles on root penetration in soil parent material (left) and compacted soils (right)

We conducted a systematic review to understand root traits and mechanisms that allow plant roots to penetrate and grow in soil parent materials. Following the widely applied protocols of PRISMA (Moher et al. 2009; Moher et al. 2015), steps were taken to minimise bias throughout the identification, selection, and synthesis of studies. ***Databases***—we used Web of Science and Scopus to ensure we examined peer-reviewed literature. ***Search string***—we developed a search string to assemble papers that identify root traits or explain the mechanisms that enable roots to penetrate and grow in soil parent materials (Fig. 1). ***Screening***—during the screening, the search string terms needed to feature in the title, abstract, or keywords for the article to be included. The search was restricted to original research papers and review articles written in English.

The search was conducted on the 5th of July 2024 and yielded 503 research articles (301 from Scopus and 202 from Web of Science). Of these, 185 duplicates were discarded, leaving 318, which were assigned to abstract screening. The key criterion used to screen the abstracts was that the abstract must mention at least one root trait or root mechanism that enables roots to penetrate and develop in soil parent materials. Following the abstract screening, 241 research articles were rejected, and 77 research articles were assigned to full-text screening. During the full-text screening, 67 research articles did not sufficiently meet the criteria above and were rejected. ***Data extraction***—A total of ten research articles were subjected to manual data extraction, and relevant data were inputted into a database. The full list of relevant data included the type of root trait(s) and mechanism(s) identified, method(s) used to measure the root trait(s) or mechanism(s), type of experiment done, the geographical location where the experiment was conducted and its climate, types of soil parent materials, degree of weathering, profile depth, plant type, its rooting system, and growth habit (Supplementary Information 1).

After full-text screening, the first search string yielded only ten relevant research articles, which was too small a pool to conduct an in-depth systematic review. To discuss root traits and mechanisms that help plants overcome mechanical impedance, we included a review of traits that promote root penetration in compacted soils due to their overlapping bulk densities and penetration resistances [(compacted soils (0.86–1.91 g cm^–3^, 0.5–2.5 MPa) (Olowolafe 2002; Grzesiak et al. 2013) and soil parent materials (1.65–2.34 g cm^–3^, 2.0—6 MPa) (Graham et al. 1997; Stephan et al. 2015)]. ***Search string***—we, therefore, developed a second search string to collect papers on root traits and mechanisms that enable roots to penetrate and grow in compacted soils rather than soil parent materials (Fig. 1). All criteria for the initial selection and screening remained the same.

The second search was conducted on the 17th of July 2024 and yielded 1,034 research articles (592 from Scopus and 442 from Web of Science). ***Screening***—In this search, we included research papers that studied compacted soils. From the search yield, 314 duplicate results were discarded, and 720 were assigned to the manual abstract screening stage. The key criterion used to screen the abstracts was that the abstract must mention at least one root trait/mechanism that enables roots to penetrate and develop in compacted soils. After the abstract screening, 507 research articles were rejected, and 213 were passed on to the full-text screening stage. During the full-text screening phase, 102 research articles did not sufficiently meet the criteria above and were rejected. ***Data extraction***—a total of 111 research articles were subjected to manual data extraction, where relevant data were inputted into a database. The full list of relevant extracted data was the same as in the first search, except for soil type, soil texture, and degree of compaction (Supplementary Information 2).

### Species studied

**Fig. 2:**
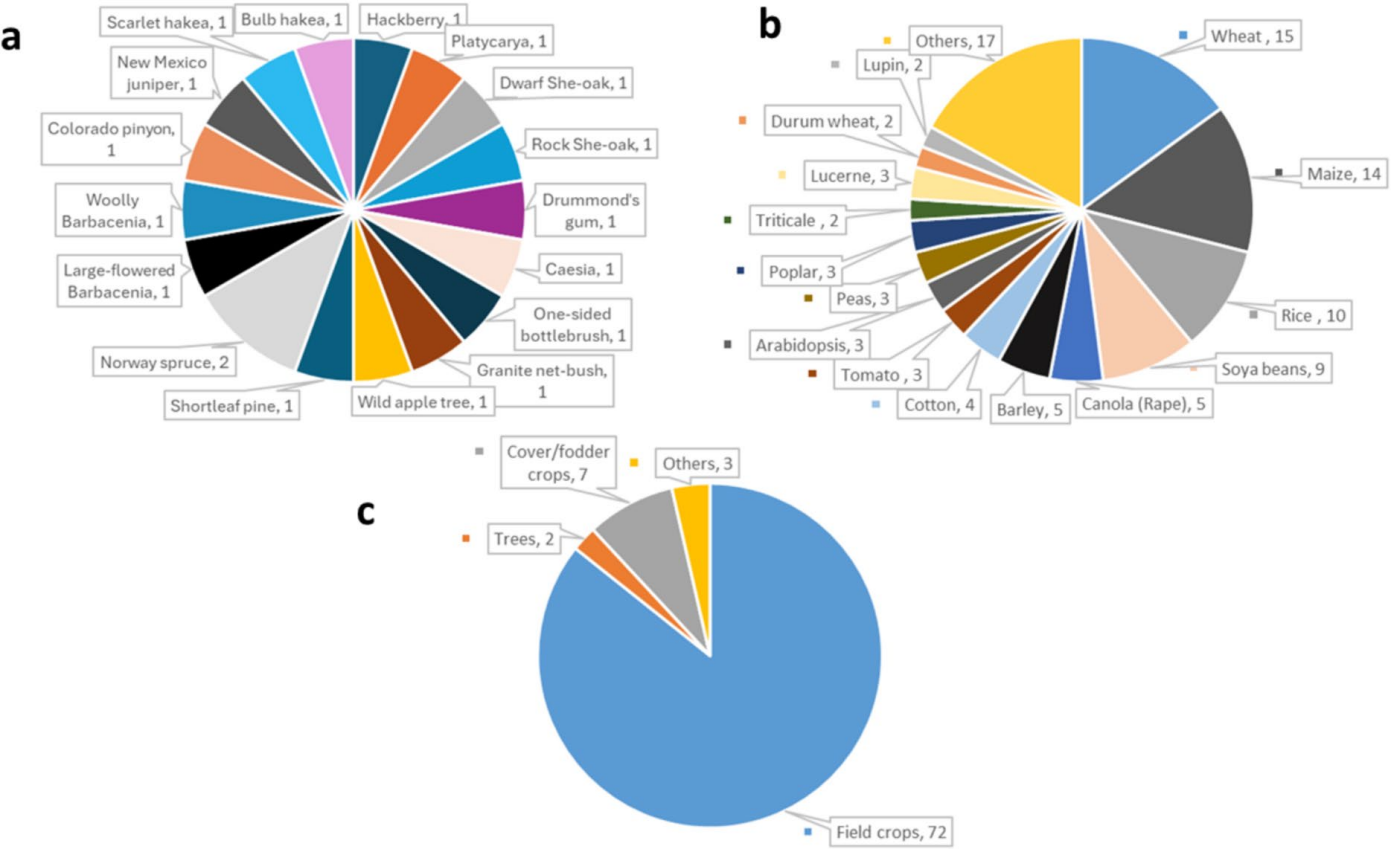
Species studied for their root traits and mechanisms under **a**) soil parent materials, **b**) compacted soil, and **c**) categories of species studied under compact soils

Under the soil parent materials search string, we noted that 18 tree species were studied in ten research articles. Most species were mentioned only once across the ten research papers reviewed. The only exception was Norway spruce (*Picea abies* L.*)*, which appeared more than once (n = 2) (Fig. 2a). Among the crops studied in relation to compacted soils, wheat was the most frequently researched (*n* = *15*). It was followed by maize (*Zea mays* L.) (*n* = *14*), rice (*Oryza sativa* L.) (*n* = *10*), and soybean (*Glycine max* L.) *(n* = *9*) (Fig. 2b). Cereal crops appear in 53 research papers (Fig. 2b). Because the species studied under compacted soils were so diverse, we grouped them into four broad categories; the common category was field crops (*n* = *72*), and the least common was trees (*n* = *2*) (Fig. 2c).

### Root types that were studied under soil parent materials and compacted soils

In soil parent materials, only tap rooting systems were mentioned (*n* = *10*), which were predominantly associated with tree species. The most frequently studied root types in studies on compact soils were fibrous (*n* = *61*) and tap rooting systems (*n* = *45*).

### Global distribution of research on root traits and mechanisms under soil parent materials and compacted soils

**Fig. 3:**
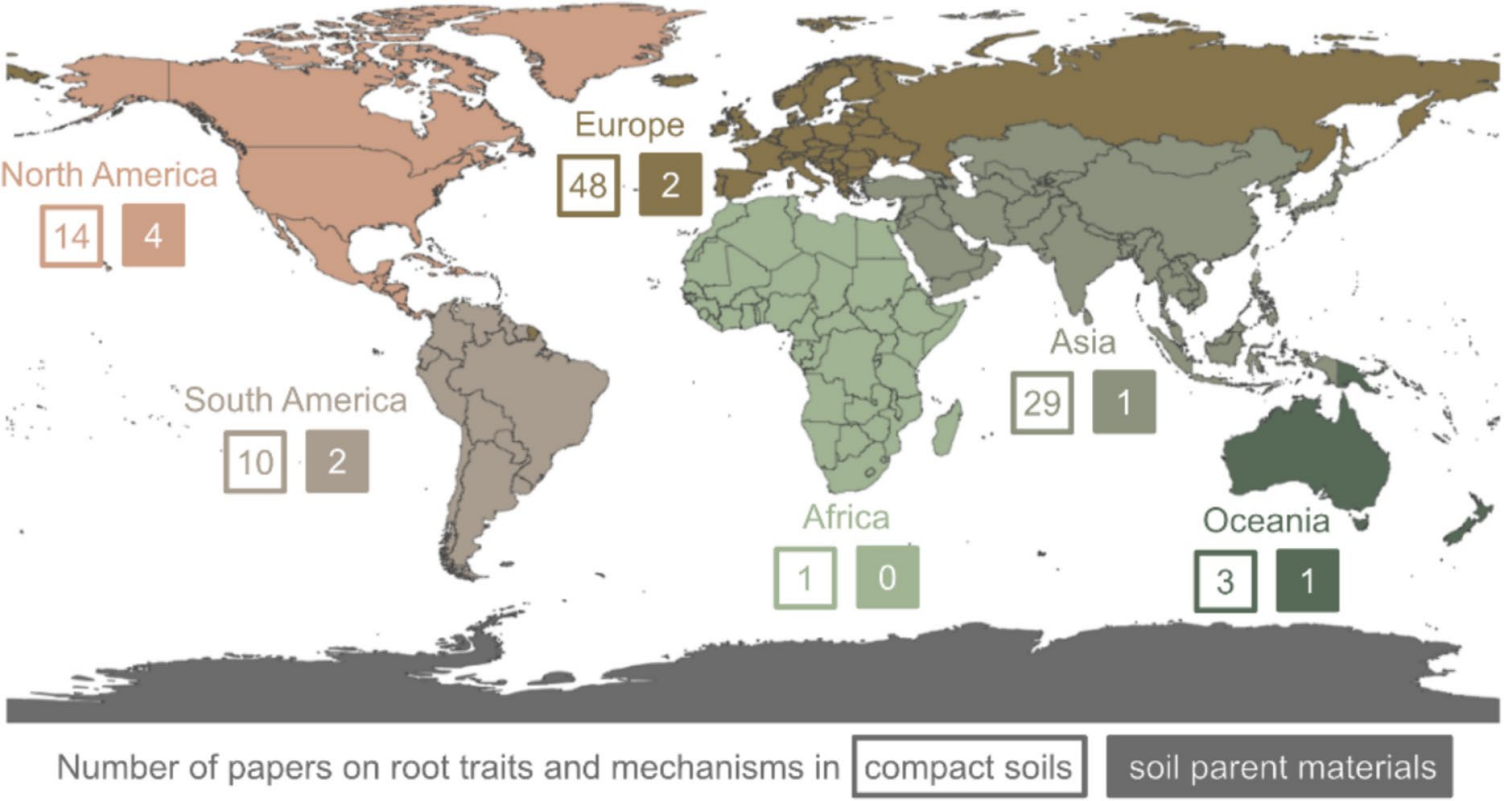
Global distribution of research on root traits and mechanisms under soil parent materials and compacted soils

The geographical distribution of the research studies helps us understand which geographical areas were represented and possible spatial relationships between soil parent materials, soil, and climate. We used the location where the experiment was carried out for original research articles and the affiliation of the corresponding author for review papers to determine the geographical distribution of studies. North America had the highest (*n* = *4*) number of research studies on root traits and mechanisms under soil parent materials, and Oceania and Asia had the lowest (*n* = *1*) (Fig. 3). Europe had the most research studies on root traits and mechanisms under compacted soils (*n* = *48*), and Africa had the lowest (*n* = *1*) (Fig. 3). The distribution shows that preliminary studies on root traits and mechanisms that penetrate soil parent material are mainly concentrated in North America, and mainly those on root traits and mechanisms that penetrate compact soils were mainly done in Europe and North America, compared to the rest of the world. This is likely because these regions have high gross domestic products and highly funded research institutes.

### Methods used to measure root traits under soil parent materials and compacted soils

**Fig. 4:**
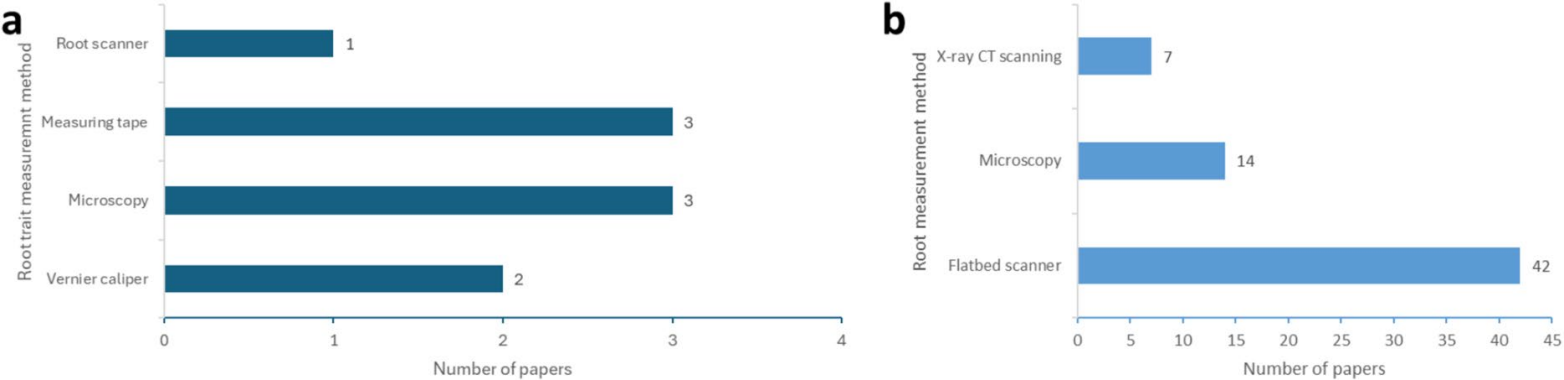
Main methods used to measure root traits under (**a**) soil parent materials and (**b**) compacted soils

Knowing different measuring methods enables us to correctly interpret the data, considering the strengths and limitations of each method. It gives the research community confidence knowing that these experiments are replicable and reproducible. In soil parent material, microscopy (*n* = *3*) and measuring tape (*n* = *3*) were used most, followed by a vernier caliper (*n* = *2*) and root scanner (*n* = *1*) (Fig. 4a). Imaging root systems using a flatbed scanner was the most common method used to measure root traits under compacted soils (*n* = *42*), followed by microscopy (*n* = *14*), and lastly, X-ray CT (*n* = *7*) (Fig. 4b).

### Types of experiments used to study root traits and mechanisms under soil parent materials and compacted soils

All experiments investigating root traits and mechanisms in soil parent material were field experiments (*n* = *9*). The most common types of experiments under compacted soils were simulated controlled (laboratory) experiments (*n* = *62*), followed by field experiments (*n* = *18*) and, lastly, controlled experiments (*n* = *7*), where the research was undertaken in controlled environments.

### Types of soil parent materials and soil textures studied

**Fig. 5:**
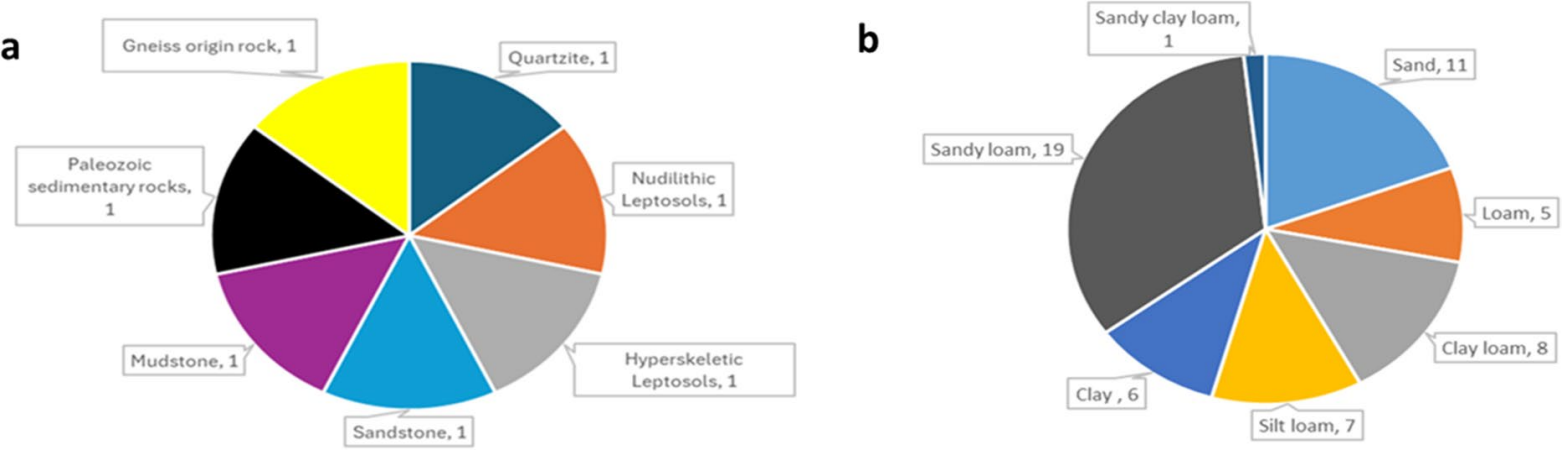
Types of (**a**) soil parent materials and (**b**) soil textures used to study root traits and mechanisms

Seven soil parent materials used to study root traits and mechanisms are illustrated in Fig. 5a. Each soil parent material was examined once, i.e., each in a single research paper (*n* = *1*). The most studied soil texture was sandy loam (*n* = *19*) and the least studied was sandy clay loam (*n* = *1*) (Fig. 5b).

**Fig. 6:**
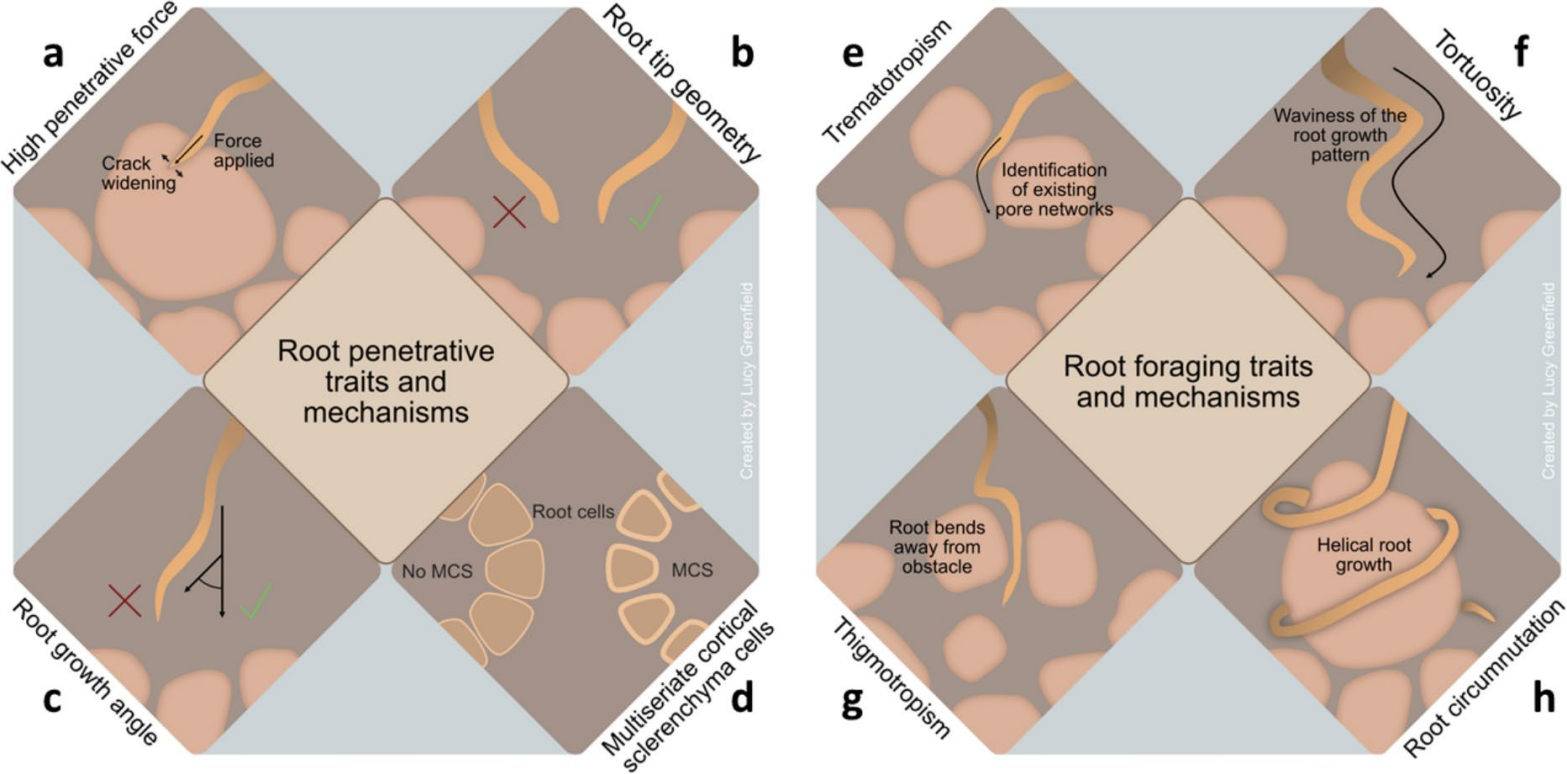
Various root traits that assist plants in penetrating soil parent materials and compacted soils include several mechanical and foraging attributes. Under the broad category of mechanical root characteristics, notable examples are **a**) high penetrative force **b**), root tip geometry **c**), root growth angle **d**) and MCS. In terms of foraging attributes, notable examples are **e**) trematotropism **f**) tortuosity **g**), thigmotropism **h**) and root circumnutation

Some root traits and mechanisms have been proven effective in overcoming the penetrative resistance of both compacted soils and soil parent materials. However, other traits may help penetrate compacted soils but not soil parent materials (Table 1). Reported root traits and mechanisms fall into three broad categories, namely those relating to root penetration of parent material, root foraging to explore better parent material, and remote exploitation of resources derived from parent material (Table 1, Fig. 6.

**Table 1:**
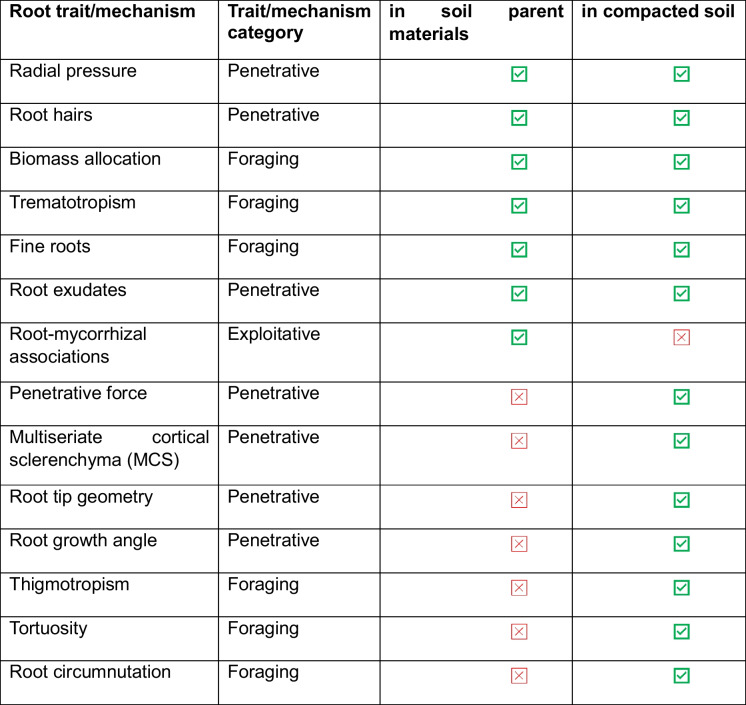
A summary of root traits and mechanisms that were reported in papers using soil parent materials and compacted soils

## Root traits and mechanisms that promote penetration

When roots encounter an area with high penetrative resistance, they have three options: a) they can stop growing altogether; b) they can penetrate the resistant layer to continue growing downward; or c) they can circumvent the obstacle by growing sideways (Correa et al. 2019). Each of these options depends on different root traits, mechanisms, and penetrative resistance, as well as the structural integrity of the growing media. Roots of commonly studied crops do not continue growing under growth media with penetration resistance beyond 6 MPa (Bengough et al. 2011). Root mechanical traits, such as penetrative force, tensile force, tensile strength, modulus of elasticity, and tensile strain, are important characteristics that influence the penetration of roots into the soil (Chimungu et al. 2015a, b).

Greater penetrative force enhances the ability of roots to use mechanical strength to penetrate and grow against mechanical resistance in their path (Fig. 6a). Colombi et al. (2017a, b) observed that roots must exert greater penetrative force when growing in compacted soils, which leads to an increased rate of penetration. Duan et al. (2023) found that rapeseed (*Brassica napus* L.) cultivars with higher concentrations of root cellulose and lignin exhibited greater root tensile force and tensile strength. These cultivars also demonstrated a stronger response in terms of maximum rooting depth and specific root length compared to those with lower concentrations of cellulose and lignin. These findings align with previous studies that indicate that stiffer roots, characterised by high tensile force and strength, are linked to greater rooting depth in compacted soil (Chimungu et al. 2015a, b; Clark et al. 2008; Lee et al. 2020). In an experiment designed to determine the minimum force required for hybrid poplar (*Populus deltoides* * *Populus. nigra*, cv ‘Soligo’) roots to buckle and deform, Bizet et al. (2016) observed that the force exerted by growing roots increased by more than 15-fold when buckling was prevented through the lateral bracing of the roots with mature, thickened cells with high turgor pressure. These authors identified the junction between the growing zone and the mature zone of the root (which contains immature cells with low turgor pressure) as a critical area of mechanical weakness that plays a key role in the root bending process. Several adaptive root traits increase the penetrative force of plants; these include multiseriate cortical sclerenchyma (MCS), a ‘sharper’ root tip geometry, a ‘steeper’ root growth angle, and the development of root hairs (Fig. 6).

### MCS

This root trait is characterised by small cells with thick walls in the outer cortical tissue (Schneider et al. 2021) (Fig. 6d). The primary function of this trait is to improve the mechanical stability of root tissue, allowing it to penetrate compacted soil more effectively using penetrative force. It is particularly beneficial in environments with mechanical impedance, as it enhances the roots’ penetrative force by increasing lignin concentration (Schneider et al. 2021). This root trait is heritable and genetically controlled (Lynch et al. 2022). Genotypes with MCS exhibited higher root lignin concentrations, increased cortical tensile strength, and enhanced root tip bending force compared to non-MCS genotypes (Chimungu et al. 2015a; Zhang et al. 2014). In genotypes that contain MCS, the thickness of the outer cortical cell walls ranged from 7.9 to 11.1 µm, with an average thickness of 9.5 µm, giving a penetration ratio above 0.8, root tip bending force above 0.9 (N), and tensile strength averaging 12 MPa (Schneider et al. 2021). In contrast, genotypes lacking MCS exhibited outer cortical cell wall thicknesses ranging from 2.3 to 5.4 µm, averaging 4.3 µm, giving a penetration ratio below 0.45, root tip bending force below 0.6 (N), and tensile strength averaging 8 MPa. In addition to the studies included within this systematic review, Zhang et al. (2025) also revealed that cellulose regulates cell stiffness and thickness, thereby influencing radial swelling of cortex cells, which enhances root growth in compacted soil. This specialised lignified outer cortical cell is commonly found in various cereal crop species, including maize, wheat, and barley (Boudet 2000; Schneider et al. 2021). The buildup of lignin and suberin in the exodermis and endodermis may also enhance the mechanical stability of root tips. Lignin and suberin also act as barriers in the exodermis and stele cell types, working together to prevent root radial water loss (Zhu et al. 2025). Schneider et al. (2021) observed that in maize, higher concentrations of lignin in the roots were associated with increased tensile strength of the root cortex, which enhances the root's penetrative force. In wheat, a positive correlation was found between the ratio of cell wall area to lumen area and the tensile strength of the root cortex. On average, the tensile strength of the root cortex was 20% greater in maize lines with MCS compared to those without MCS and 28% greater in wheat lines with MCS compared to those without. Furthermore, maize genotypes with MCS demonstrated 48% greater ability to penetrate a hard wax layer than those without MCS. In comparison, wheat genotypes with MCS exhibited an average of 62% greater penetration ability through the same type of barrier compared to their counterparts without MCS.

The formation of MCS is primarily triggered by ethylene, which is released by roots and builds up around the roots in response to soil compaction (Pandey et al. 2021; Schneider et al. 2021). This accumulation of ethylene restricts root growth in compacted soils. Therefore, it is proposed that ethylene accumulation serves as an early warning signal for roots to avoid compacted soil (Pandey et al. 2021). While MCS is valuable for root penetration in compacted soils, its contribution to how effectively deep-rooted species or trees might be able to penetrate soil parent material has not been thoroughly studied.

### Root tip geometry

The shape of a root tip influences its ability to penetrate the soil (Fig. 6b). Colombi et al. (2017a) found that certain wheat varieties capable of penetrating compacted soils have a 'sharper' root tip shape. In contrast, varieties with a 'rounder' root tip shape struggle to penetrate compacted soils. The root tip shape is regulated by ethylene, which causes root tips to thicken under mechanical impedance; ethylene-insensitive mutant roots retained their 'sharp' shape instead of becoming 'thickened' when exposed to compacted soil (Pandey et al. 2021). Additionally, a lower root tip radius-to-length ratio is associated with a higher root elongation rate, while root diameter does not seem to influence root elongation based on genetic differences (Colombi et al. 2017a; Vanhees et al. 2020). A smaller root tip radius-to-length ratio helps reduce penetration stress, allowing for greater root elongation rates in compacted soil (Colombi et al. 2017a). The research above indicated that the geometry of the root tip is a crucial factor influencing root penetration stress and root elongation in compact soils. Therefore, the shape of the root tip should be considered when selecting crop varieties that can tolerate compacted soil. Currently, no studies have explored the extent to which root tip geometry affects a crop’s ability to penetrate soil parent materials. Future investigations could focus on crop varieties with a ‘sharper’ root tip shape as potential candidates for cultivation in shallow soils.

### Root growth angle

The angle at which a root grows in relation to a soil layer, referred to as the "root growth angle" (measured in degrees from the vertical), influences root elongation, the volume of soil available for the roots to search for water and nutrients, and whether a plant develops a shallow or deep root system (Correa et al. 2019). At a given level of soil compaction, an increase in the root growth angle (i.e., a steeper angle) allows a greater proportion of roots to penetrate compacted soil (Fig. 6c). For example, in wheat, steeper growth angles enhance the ability to penetrate compacted soils (Whalley et al. 2013). Research on root growth angle has mainly focused on food crops cultivated in compacted soils, while the root angles of trees, which are crucial for promoting deep rooting, have received less attention. Crops that exhibit steeper rooting angles may be better adapted to thrive in shallow soils. Breeding programmes that aim to develop varieties with steep-rooted characteristics could lead to crops with roots capable of penetrating deeper beyond the soil into the soil parent material.

### Root hairs

Root hairs are unbranched extensions of root epidermal cells. Their primary function is to increase the root's surface area, enhancing the absorption of water and nutrients (Evert 2006). Additionally, root hairs help anchor the root tip in the soil, allowing the expanding tissues of the root tip to penetrate further into unexplored soil. Anchorage is accomplished through several mechanisms: the cumulative friction between soil particles and the developing tissues behind the elongation zone (Bengough et al. 2011). According to Bengough and Mullins (1990) and Bengough et al. (2011, 2016), the anchorage of the root axis may facilitate the root penetration from a looser to a denser layer. Root hairs can provide sufficient tensile strength to anchor root tips by providing rigidity and helping to resist root tip displacement (Bengough et al. 2011). Root hairs might enable growing roots to attach themselves firmly to the soil pore walls and penetrate further into the surrounding soil layers. Haling et al. (2013) found that barley genotypes with root hairs possess an advantage when it comes to compact soil layers (1.7 g cm^–3^) compared to genotypes that lack root hairs, as root hairs anchor root tips. In addition to the studies included within this systematic review, Kong et al. (2024) also observed that root hairs enhance root penetration ability at bulk densities up to 1.4 g cm^–3^ in rice root hair mutants by increasing the anchorage of root tips to their surrounding soil. This assertion is further supported by Bengough et al. (2016), who demonstrated that a hairless maize mutant (*rth3-3*) exhibits a lower penetration rate than its wild-type counterpart with root hairs under conditions of soil compaction.

In contrast to studies that show how root hairs can help penetrate compacted soils, Bailey et al. (2002) found that root hairs, unlike lateral roots, do not contribute to the overall anchorage of the plant. Additionally, Hoffmann and Jungk (1996) also noted that at high bulk density (1.7 g cm^–3^), the number of root hairs per unit length of root decreased. More studies are needed to investigate root hair formation under mechanical impedance, particularly in soil parent materials that are more compact and consolidated compared to typical compact soils lacking transitional zones. An example of a follow-up research question is: Can root hairs enhance root penetration into the saprolite?

### Root exudates

Root systems release low molecular weight compounds, mucilage, detached root cap cells, and tissue debris during plant growth. One of the key functions of sugar-rich mucilage exuded by roots is to form a protective sheath around the roots, which reduces friction at the root-soil interface, enabling deeper root penetration (Bengough and Mckenzie 1997; Carter et al. 2019; Liu et al. 2019). Root exudates are primarily released from the root apex (Groleau-Renaud et al. 1998; Pawlik et al. 2016). As the number of root tips increases, the overall volume of exudation also increases (Atwell 1990). The volume and composition of root exudates are significantly influenced by root branching and structure. Secretions from roots that encounter resistance may be influenced by changes in root structure (Atwell 1990), while carbon exudation can increase as the root's diameter and surface area increase (Groleau-Renaud et al. 1998).

Additional studies indicate that increased external impedance leads to the shedding of root cap cells and promotes the exudation of root mucilage. This lubrication reduces the friction between roots and soil, aiding in root penetration (Bengough et al. 2011; Bengough and Mckenzie 1997; Iijima et al. 2003; Mckenzie et al. 2013; Piccoli et al. 2021). This mechanism also helps roots resist mechanical stress (Okamoto and Yano 2017), lowers root-soil friction, thus promoting root elongation (Oleghe et al. 2017). Oleghe et al. (2017) measured root exudates under soil compaction stress and observed that, with 600 kPa compression, increasing the amount of exudate from 0 to 1.85 mg g^−1^ reduced penetration resistance by 77% in sandy loam soils. These root exudates are positively correlated with the maximum root depth in compacted soil. This suggests that increased root exudation, stimulated by mechanical impedance, can help roots elongate into deeper soil layers. The pathways provided by biopores during root elongation (Hinsinger et al. 2009; Oleghe et al. 2017), along with the higher density of fine roots facilitated by greater root exudation, play a positive role in root penetration and elongation in compacted soil (Duan et al. 2023). If increased root exudate production leads to a modification of the rhizosphere's physical environment by loosening soil particles through lubrication, it can reduce the friction encountered by the root tips during penetration (Chen et al. 2022; Naveed et al. 2017). Root exudates also alter the chemical environment of the rhizosphere by stimulating microbial activity, which contributes to pore formation and enlarging existing ones, which is another factor enhancing the ability of roots to penetrate the soil (Badri and Vivanco 2009; Boeuf-Tremblay et al. 1995).

Tree roots also have a significant impact on their surrounding environment through the production of root exudates in the rhizosphere (Hinsinger 1998). Deep roots contribute to the physical and chemical weathering of mineral materials through exerting radial pressure and the production of root exudates, playing an essential role in soil formation (pedogenesis) (Richter and Markewitz 1995). While most research on root exudates has concentrated on topsoils, it is important to acknowledge that similar processes likely occur in deeper soil layers (Richter and Markewitz 1995). However, the specific biogenic effects of deep root exudates remain unclear. Further studies that compare exudate and rhizosphere chemistry in uncompacted soils, compacted soils, and soil parent materials may provide better insights into how these exudates facilitate root penetration in soil parent material.

### Radial pressure

Root axes often thicken as they penetrate compacted soils. Typically, this increase in root diameter is due to an increase in cell sizes rather than an increase in the number of cells within the root (Pritchard 1994). Ethylene-dependent responses to soil compaction result in reduced root elongation and increased radial swelling (Huang et al. 2022). Ethylene accumulation around the roots triggers the production of abscisic acid, which, in turn, promotes root radial expansion in cortical cells (Kirby and Bengough 2002; Pandey et al. 2021), leading to an increase in the root's penetrative force. Ethylene-insensitive mutants do not exhibit these compaction-induced root growth responses (Frene et al. 2024). Various studies on field crops have shown that root diameter increases in compacted soil (Correa et al. 2019; Huang et al. 2022; Pritchard 1994). The enlargement of the main roots is believed to enhance mechanical properties, providing greater penetrative force that supports improved axial root growth. The increased radial pressure enables roots to exert growth pressure, displacing soil particles and overcoming friction, which allows them to elongate through the soil (Clark et al. 2003). As a result, thicker roots are better at penetrating compacted soil (Correa et al. 2019) because they can resist buckling and deflection (Jin et al. 2013; Lipiec et al. 2003). Helliwell et al. (2019) noted an increase in root diameter (1.3 mm) in compacted soils (1.5 g cm^–3^) compared to (0.82 mm) in uncompacted (1.2 g cm^–3^) loamy soils. Additionally, Materechera et al. (1992) conducted a study on several monocot and dicot species, including oats (*Avena sativa* L. cv. Dolphin), ryegrass (*Lolium rigidum* L. cv. Wimmera), safflower (*Carthamus tinctorius* L. cv. Gilla), wheat (*Triticum aestivum* L. cv. Kite), barley (*Hordeum vulgare* L. cv. Galleon), fava bean L. (*Vicia faba* cv. Fiord), lupine (*Lupinus angustifolius* L. cv. Gungurru), and pea (*Pisum sativum* L. cv. Early Dunn). They found that a higher proportion of thicker roots is linked to greater root penetration in compacted soil.

In tree species, the radial pressure (sideways) exerted by root systems can reach up to 0.91 MPa, while axial pressures (downwards) may reach as high as 1.45 MPa (Bennie 1991). These pressures are sufficient to break up bedrock. The radial pressure from root growth causes the widening of joints and maintains direct contact with the interior surfaces of those joints (Pawlik et al. 2016). As tree roots increase in length and girth, they gradually split the rocks apart (Jerin 2019). Phillips (2015) demonstrated that approximately 90% of the trees studied in research conducted on limestone bedrock showed evidence of root penetration having caused the widening of joints in the bedrock, both horizontally and vertically.

Research shows that plants apply root radial pressure differently depending on the type of growth medium. In soil parent materials, root radial pressure is effective in widening cracks and breaking rocks apart. In compacted soils, radial pressure displaces soil particles and overcomes friction by exerting pressure sideways and creating fissures. Future research could investigate ethylene-insensitive mutants in shallow soil to determine if their roots can successfully penetrate and grow in saprolite.

## Root foraging traits and mechanisms

Foraging refers to the process by which roots explore their environment to find optimal conditions for growth. When roots encounter obstacles, such as rock fragments in the soil, they typically avoid sudden mechanical stress by seeking out better-growing conditions nearby (Adak et al. 2019). Adak et al. (2019) noted that foraging length (the distance roots grow in length until they reach areas with less resistance) plays a crucial role in navigating around areas of mechanical impedance. The foraging strategies employed by roots are influenced by soil textures, leading to diverse root architectures and physiological responses in plants (Kolb et al. 2017). There are various foraging mechanisms and traits, which include root thigmotropism, tortuosity, circumnutation, below-ground biomass allocation, trematotropism and the development of fine roots (Fig. 6).

### Thigmotropism

When a root tip encounters a barrier, it quickly bends away from the obstacle in a reaction known as thigmotropism (Massa and Gilroy 2003). This is followed by a second bending movement along the direction of gravity, resulting in a step-like growth pattern (Fig. 6g). Both bending responses depend on asymmetrical cell expansion in the root elongation zone (Li and Jia 2022). Roots exhibit an ability to change their growth direction, showcasing flexibility that may be associated with the substantial amount of cortical tissue found in them (Belzunce et al. 2008). Bending has been observed in roots that encounter a soft upper layer and a hard lower layer. In these instances, roots respond by bending when they reach the harder layer (Yamamoto et al. 2008; Yan et al. 2017, 2018). Researchers have suggested that a zone of "mechanical weakness" located between the growing and mature zones of the root is necessary for the bending process (Bizet et al. 2016).

### Tortuosity

Another root foraging behaviour is root tortuosity, i.e., the waviness of the root growth pattern (Popova et al. 2016) (Fig. 6f). The degree of tortuosity in a root system is influenced by both soil bulk density and soil texture. It can be quantified by comparing the length of the primary root to the vertical depth of the root system; this ratio reflects how much longer the actual root path is compared to the shortest possible path (Tracy et al. 2013). Roots commonly navigate through cracks and holes in the soil but move in a bending motion as they grow (Correa et al. 2019). Most compacted soils have some form of preferential paths, and roots typically thrive in these areas. Roots are flexible structures that tend to follow paths of least resistance. By following weak planes between soil particles, roots may experience reduced frictional resistance, facilitating their penetration into the soil. Additionally, tortuous root growth may occur as roots conform to the surfaces of soil aggregates (Lipiec et al. 2003). Thigmotropism and tortuosity may appear similar, but they are distinct phenomena that occur in plant roots. Thigmotropism is triggered by physical contact, leading to directional growth responses as roots navigate through their environment. In contrast, tortuosity is affected by various factors and results in a more intricate, wavy growth pattern that optimises resource acquisition.

Tracy et al. (2012) observed that the interaction between bulk density and soil type significantly affected the tortuosity of tomato (*Solanum lycopersicum* L. cv Ailsa Craig) plant roots. Specifically, the values for root path tortuosity were higher in plants grown in compacted soil (1.6 g cm^−3^) compared to those in uncompacted soil (1.2 g cm^−3^). This indicates that soil compaction increases root tortuosity. The introduction of non-destructive visualisation techniques, such as X-ray CT scanning (Ghosh et al. 2023), has enabled the study of root tortuosity in compacted soils using mesocosms. Future research could apply these same techniques to examine root tortuosity in deep-rooting species within soil parent material, as there is currently no documented evidence of such studies in the literature.

### Root circumnutation

The helical movement of growing root tips is a commonly observed behaviour in plants that allows them to navigate around mechanical obstacles (Fig. 6h), thereby aiding their establishment in stoney soil (Leuther et al. 2024; Taylor et al. 2021). Root nutation is considered beneficial for reducing soil resistive forces (Ruiz et al. 2017). The intensity of root circumnutation, characterised by the amplitude and frequency of this helical movement, is influenced by both genetic and environmental factors (Leuther et al. 2024). In rice (*Oryza sativa* L. cv Dongjin), researchers have identified genotypic differences in the amplitude (Taylor et al. 2021) and frequency (Inoue et al. 1999) of circumnutation. Additionally, studies have shown that lentil (*Lens culinaris* cv Peridot) plants increase their circumnutation amplitude in response to greater mechanical impedance (Martins et al. 2020). Furthermore, the stiffening of the root growth zone due to cell shortening (Croser et al. 2000; Liu et al. 2022) enables roots to exert a greater radial force on the soil, which may contribute to an increase in circumnutation amplitude. With the advent of X-ray CT technologies (Ghosh et al. 2023), future research on root circumnutation will produce deep physiological insights, particularly concerning what triggers root circumnutation rather than tortuosity or thigmotropism or vice versa.

### Biomass allocation

Biomass allocation patterns are influenced by ontogenetic drift, which may decrease or increase the level of phenotypic plasticity over time (Evans 1972). McConnaughay and Coleman (1999) demonstrated that some annual species (*Chenopodium album*, *Polygonum pensylvanicum*, and *Abutilon theophrasti*) showed remarkable plasticity in growth rates and significant amounts of ontogenetic drift in root: shoot biomass ratios across different conditions (water, light, and nutrients). This is consistent with optimal partitioning theory. There is evidence of ontogenetic drift in biomass allocation when plants encounter mechanical impedance stress, some plant species tend to allocate more biomass to their root systems as a strategy to cope with mechanical impedance (Fig. 8). High root biomass means a high root surface area, which allows plants to explore a larger soil volume and find areas with lower mechanical resistance. Several studies on root growth have been conducted in controlled experiments, demonstrating that biomass allocation can be influenced by root mechanical impedance.

In compacted soils, Duruoha et al. (2007) observed that root biomass (measured as root dry matter) increased at higher levels of soil compaction (1.60 g cm^−3^) compared to lower compaction levels (1.20 g cm^−3^). In addition, Asif et al. (2023) found that *Eucalyptus camaldulensis* Dehnh. exhibited a significantly higher root-to-shoot ratio (1.1) at the highest bulk density tested (1.80 g cm^−3^) compared to 0.6 at the lowest bulk density tested (1.30 g cm^−3^). This indicates that *E. camaldulensis* has a greater ability to allocate resources to root development in response to compaction than other studied species, such as *Albizia lebbeck* L., *Vachellia nilotica* L., and *Ziziphus mauritiana* L. Bingham et al. (2010) observed that high soil compaction (1.80 MPa) increased the allocation of biomass (0.20 g) to the root system in barley compared to 0.13 g under lower compaction levels (0.56 MPa). This increase in root diameter may indicate a greater investment of resources to strengthen roots in response to mechanical resistance, enhancing the roots' penetrative force. While previous studies have demonstrated that investing heavily in below-ground biomass can help overcome penetrative resistance, it remains unclear whether this investment negatively impacts above-ground biomass or potentially reduces yields in food crops.

In soil parent materials, research has demonstrated that *Hakea* species endemic to shallow soil, granite outcrop communities in southwestern Australia, increase their root surface area and foraging length to enhance their chances of accessing fissures in the underlying bedrock. In a seedling pot experiment, these shallow-soil endemic *Hakea* species allocated a larger proportion of their biomass to roots and explored a wider area of soil compared to *Hakea* species from habitats with deeper soils (Poot and Lambers 2008). Ma et al. (2020) investigated the adaptation strategies of two common plant species: the deciduous tree *Platycarya longipes* and the evergreen shrub *Tirpitzia ovoidea*. These species were studied in two contrasting habitats: a shallow soil and a nearby deep soil. The researchers observed that both species exhibited extensive lateral root expansion and a high root-to-canopy ratio in the shallow soil layers rather than deep root penetration. Specifically, the trees’ wide horizontal root spread in the soil-limited habitat was characterised by a slow rate of root tapering and bending. These root responses to rocky substrates are consistent with common responses observed in terrestrial plants growing in compacted sediments or rocky conditions (Clark et al. 2001; Fageria et al. 2006; Materechera et al. 1991, 1992; Tracy et al. 2012).

### Trematotropism

When roots encounter compacted soil structures, a critical mechanism that facilitates deeper rooting is the capacity to identify and take advantage of existing pore networks (Fig. 6e). This ability is known as trematotropism (Atkinson et al. 2020). Roots can navigate around compacted soil layers by utilising macropores, which are soil cavities larger than 75 µm. In compacted soils with a bulk density of 1.6 g cm^−3^, Atkinson et al. (2020) showed that 68.8% of the interactions between roots and macropores led to the colonisation of these pores by wheat roots. In contrast, only 12.5% of such interactions occurred in relatively loose soil with a bulk density of 1.2 g cm^−3^ (Atkinson et al. 2020). The results suggest that colonising macropores was a crucial strategy for plants growing in the compacted subsoil. Colombi et al. (2017a, b) investigated the interactions between the roots of soybean, wheat, and maize with artificial macropores using X-ray CT. They observed that the roots of all three species actively grew towards the artificial macropores, specifically, maize roots predominantly grew into the macropores. Artificial macropores in compacted soil enabled all three species to compensate for reduced early growth in later developmental stages. The various types of root-macropore interactions showed that macropores provide a path of least resistance and a source of oxygen. As a result, crop productivity on compacted soils increased and became comparable to that of uncompacted soils.

In natural field conditions, channels in the soil typically take the form of cylindrical biopores, such as those created by earthworm tunnels or the decay of previous root systems (Atkinson et al. 2020). Stirzaker et al. (1996) discovered that barley thrived to a greater extent in compacted soils (1.8 g cm⁻^3^) that contained a network of narrow biopores formed by lucerne (*Medicago sativa* L.) or ryegrass (*Lolium perenne* L.) roots, compared to soils with larger artificially constructed pores. They noted that roots benefitted when these biopores were filled with peat. Additionally, Whalley et al. (1999) found that carrot (*Daucus carota* L.) seedling roots were not adversely affected by mechanical impedance (0.75 MPa) in sand culture systems. This was due to the fine carrot roots being small enough to easily elongate through the pores of the sand.

Tree roots almost certainly exploit structural pores at great depths. Jackson et al. (1999) show that deep rooting (> 10 m) is common in tree species in natural environments. This is likely to be the factor that allows very deep rooting in sandstone, where Canadell et al. (1996) report roots to a depth of 53 m. The mechanisms by which roots locate soil pores are not well understood. Given that mechanical impedance typically increases with depth, exploring this topic could provide significant insights. The chance of roots encountering a pore is influenced by both the branching pattern of the root system and the density, distribution, and connectivity of the pores in the soil.

### Fine roots

Fine roots are defined as those with a diameter of less than 0.25 mm (Cubera et al. 2009). Some soil parent materials, such as sandstone, are characterised by small pores (50 to 400 μm, Zhao et al. 2015); only fine roots can penetrate these pores. Fine roots enhance the root surface area per unit mass (Eissenstat 1992). Fried et al. (2018) found that the traits of fine roots are related to the overall characteristics of the root system. For instance, fine root length facilitates plant growth in compacted substrates through micropores (1 µm—10 µm, Zhao et al. 2015). Several authors have noted that, contrary to the idea that higher fine root density enhances a plant’s ability to forage micropores in compacted layers, fine root density often decreases in more compacted soils. For instance, Cubera et al. (2009) found that fine root growth in holm oak (*Quercus ilex* L.) seedlings was significantly reduced in areas with localised higher bulk density layers within heterogeneous soil. This observation aligns with previous studies on other forest tree species; for example, fine root density in mature blue gum (*Eucalyptus globulus* L.) sharply decreased in layers of higher bulk density, then increased again when the bulk density dropped (Gaitán and Penón 2003). Alameda and Villar (2012) indicated that soil compaction adversely affects the proportion of fine roots in narrow-leaved ash (*Fraxinus angustifolia*). Additionally, Correa et al. (2022) observed that fine roots under compacted soils (1.8 g cm^−3^) were 54% shorter than in less compacted soils (1.4 g cm^−3^).

On the other hand, in tree species, fine roots grow along bedding planes and increase the size of cracks in rocks by exerting radial pressure (Nascimento et al. 2021). Schwinning (2020) observed that fine roots often remain confined within joints and fractures. This behaviour allowed the roots to encircle quartzite fragments, thereby enhancing the surface contact between the roots and the rock. Additionally, Wang et al. (2023) noted that high gravel content in weathered rock layers could hinder root penetration and growth. However, small gaps between gravel and soil particles allowed fine roots to eventually grow and form a network around the gravel. The formation of fine roots in weathered rock layers may vary depending on factors such as vegetation species, rock type, and the level of mechanical resistance. Further research examining different root diameter classes under simulated mechanical impedance, using various growth media, and the use of X-ray CT could offer valuable insights into how fine roots overcome mechanical impedance.

## Remote exploitation of resources in soil parent materials through root-mycorrhizal associations

The significance of arbuscular mycorrhizal symbiosis for the survival and growth of many plant species is widely acknowledged (Jeffries et al. 2003). The ability of plants to form mycorrhizal associations may be crucial for their capacity to acquire resources from compacted soils and parent materials (Bornyasz et al. 2005). Although mycorrhizal associations may not be directly associated with root penetration, they improve the capacity of roots to acquire resources from the parent material and, to a lesser extent, participate in rock weathering, thereby reducing their penetrative resistance. Egerton-Warburton et al. (2003) and Bornyasz et al. (2005) demonstrated that roots of oaks and chaparral shrubs utilise mycorrhizal fungi to extract water and minerals from bedrock. Their research focused on the potential of different arbuscular and ectomycorrhizal (EM) hyphae to access these resources, particularly during drought conditions. EM hyphae are much narrower than fine roots, measuring between 2 and 10 µm, and can grow up to one metre in length (Allen 2007). Hyphae can, therefore, perform functions that some roots cannot, such as penetrating deep into the rock matrix through micropores too narrow for fine roots to access and creating numerous pathways for water to flow from the surrounding rock to plant roots. EM fungal hyphae can develop additional structures called rhizomorphs, which extend from the roots into the soil by as much as 10 cm (Taylor et al. 2009).

EM fungal hyphae associated with tree roots can penetrate soil pores that are smaller than 5 to 20 μm (Taylor et al. 2009). This association with fungal hyphae is an effective strategy for plant roots to expand their capacity for biochemical weathering. Additionally, EM fungi function as biosensors, allowing them to distinguish between different particle sizes and mineral compositions (Leake et al. 2008). This capability suggests that EM fungi can engage in selective rock weathering through fungal exudates (Leake et al. 2008). All these properties make mycorrhizal fungi associated with tree roots a very effective driver of the chemical alteration of rocks and minerals, paving the way for rooting penetration in the partially weathered rock. In the reviewed literature, this is the only root mechanism that distinguishes a soil parent material-penetrating ideotype from a compact soil-penetrating one. Although many crops can form mycorrhizal associations (Noceto et al. 2021), no research links this association to their ability to exploit compact soils.

## Plant ideotype under mechanical resistance

A crop ideotype is an idealised structural model that combines specific morphological or physiological traits to maximise yield quantity and quality within a defined environment (Donald 1968). The ideotype approach primarily focuses on defining a theoretically efficient or ideal plant type by focusing on all its component traits. One possible strategy for optimising crop productivity in shallow soils is the development of crop cultivars specifically adapted to these conditions. Crop modelling has become an essential tool in supporting plant breeding (Rötter et al. 2015) by helping to design ideotypes, or "model plants", for different cultivation environments (Dingkuhn et al. 2015; Rötter et al. 2015). Here, we suggest a plant ideotype with specialised rooting traits adapted for growth in shallow soils.

### A soil parent material penetrating ideotype

**Fig. 7:**
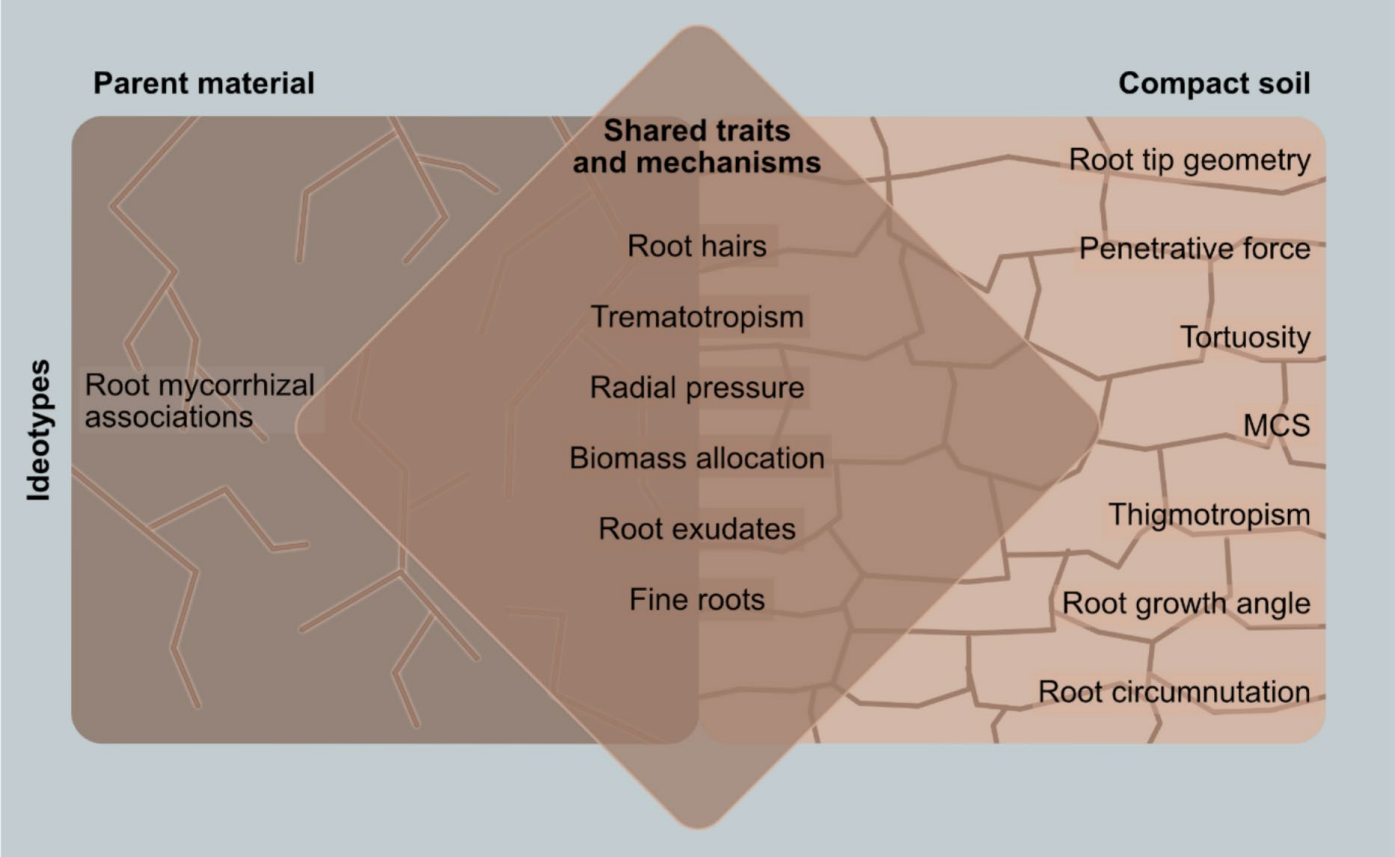
Key similarities and differences between the parent material penetrating ideotype and a compact soil ideotype

A model plant that can grow in parent material will have a combination of all the root traits and mechanisms that enable plants to penetrate, explore, and acquire resources from soil parent materials. The discussed root traits and mechanisms include root hairs, trematotropism, radial pressure, biomass allocation, root exudates, fine roots, and root mycorrhizal associations (Fig. 7). While many root traits and mechanisms overlap between parent material penetrating and compact soil penetrating ideotypes (Fig. 7, Table 1), we found no evidence in the reviewed literature on the use of penetrative force, MCS, root tip geometry, growth angle, thigmotropism, tortuosity, or root circumnutation in penetrating parent materials. Although these crop root traits were not studied in relation to soil parent material, most varieties that can penetrate compact soils appear to possess the relevant traits and mechanisms to penetrate parent materials. Therefore, we hypothesise that traits and mechanisms such as penetrative force, root biomass allocation, fine roots, root exudates, trematotropism, tortuosity, and radial pressure can enable compact soil-penetrating crops to penetrate soil parent material. Soil parent material, particularly at the soil–parent material interface, is characterised by structural weaknesses such as fissures, fault lines, cracks, interconnected pores, and is highly weathered. These features can be readily exploited by the root traits and mechanisms mentioned above (Nascimento et al. 2021; Pawlik et al. 2016; Wang et al. 2023). A crop with lignified roots growing at a steeper root angle and ‘sharper’ root tip geometry with high penetrative force may be able to penetrate softer layers (when compared to unweathered bedrock), such as highly weathered saprolite.

**Fig. 8:**
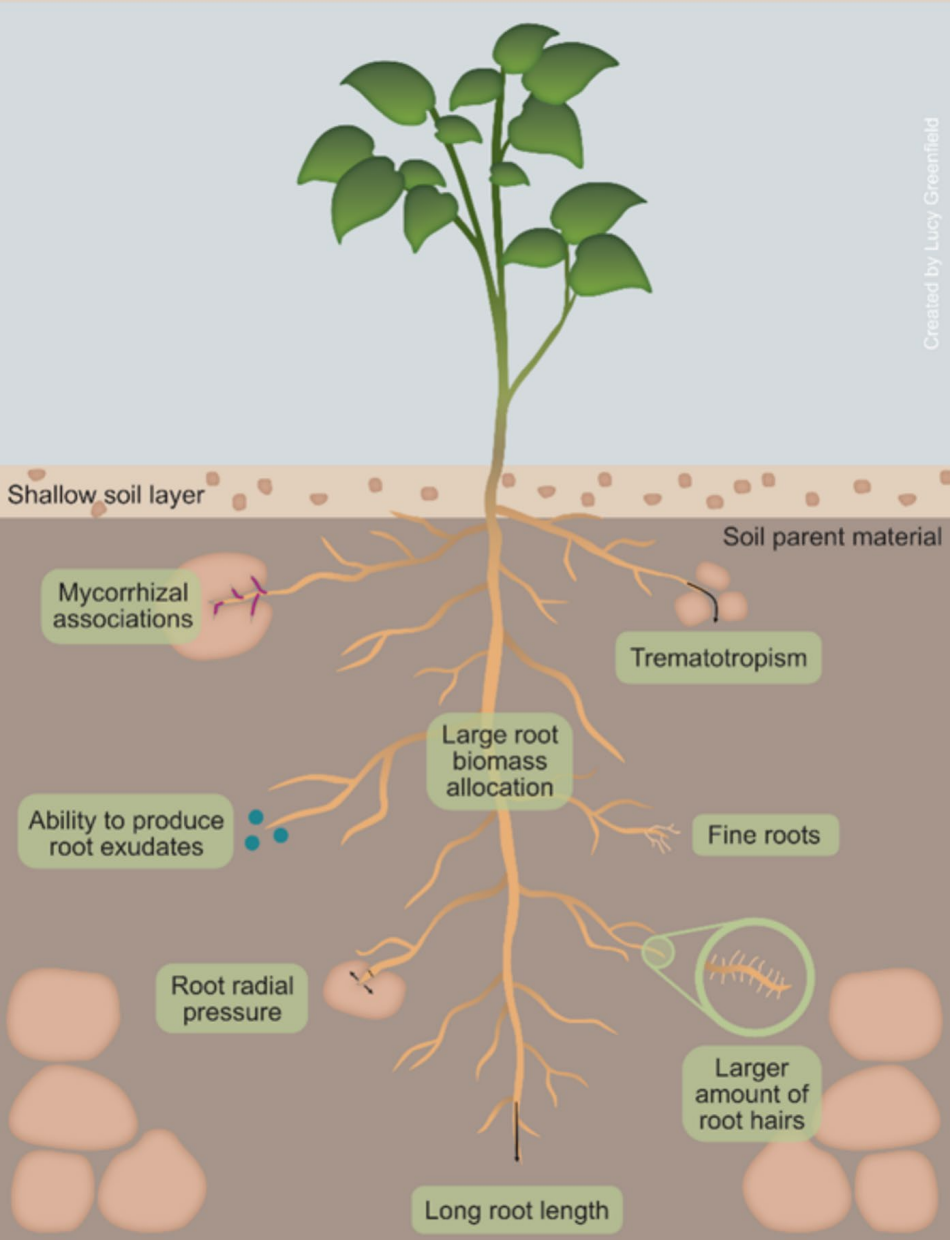
Different root traits and mechanisms that assist plants in penetrating the soil parent materials

Typically, most crop varieties are bred to thrive in well-drained, deep soils; this may explain the scarcity of studies on food crops that penetrate the parent material. These overlapping traits and mechanisms (Fig. 7) can serve as a starting point for developing a soil parent material penetrating ideotype. Several crops have been heavily studied and proven to penetrate compact soils; these include, but are not limited to, specific varieties of wheat (Colombi et al. 2017a), barley (Haling et al. 2013), MCS maize genotypes (Schneider et al. 2021), and rapeseed (Duan et al. 2023. Barley and maize varieties with root hairs have been found to penetrate compact soils better than their hairless mutant counterparts (Bengough et al. 2016; Haling et al. 2013). Bingham et al. (2010) also noted that barley tends to have a higher root biomass under compaction stress. MCS maize and wheat genotypes have thicker cortical cells, which increase root penetrative force (Boudet 2000; Schneider et al. 2021). Rapeseed has highly lignified root cells that exhibit greater root tensile force to overcome soil compaction (Duan et al. 2023). These root mechanisms and traits can be useful in penetrating soft parent material like highly weathered saprolite (Fig. 8).

## Limitations of the study

A major limitation of this review paper was the scant literature on root traits and mechanisms that enable plants to penetrate soil parent materials (*n* = *10*) (Fig. 1), primarily conducted in North America and Europe. However, these studies provided vital information that can be used to make inferences in understudied areas with similar lithologies or shallow soils. The studies also provided crucial insights for comparing root traits and mechanisms that enable plants to penetrate compact soil, as opposed to those that facilitate roots in penetrating soil parent materials. Furthermore, the studies in compact soils assist us in hypothesising which root traits and mechanisms might be useful in penetrating soil parent materials. The striking similarities in root traits and mechanisms (Fig. 7, Table 1) provide a foundation for selecting potential crops for cultivation in shallow soils. There is an opportunity to revisit this topic in the future as research articles studying root traits and mechanisms for penetrating soil parent materials in understudied regions increase.

## Future studies

Future studies can focus on the performance of these compact soil-penetrating crop varieties in shallow soils where their roots will be exposed to the parent material beneath. Further genotypic improvement of these crop varieties through breeding can result in an ideotype with root phenotypes suitable for penetrating soil parent material in shallow soils. This is the primary goal of this systematic review: to establish a foundation for a healthy, sustainable, and resilient crop production in shallow soils, where crops can grow in soil parent materials. Other future studies can focus on root penetration in different lithologies and at varying levels of weathering.

## Conclusion

A variety of root traits and mechanisms enable plants to penetrate, explore, and acquire resources from soil parent materials. These include increased root radial pressure, high root biomass investment, development of fine roots, root trematotropism, formation of mycorrhizal associations, presence of root hairs, and production of root exudates. All the above-mentioned root traits and mechanisms have also been shown to help plants overcome compacted soil, except for the formation of mycorrhizal associations. The ability to form mycorrhizal associations for nutrient and water acquisition in soil parent material has been extensively studied in deep-rooted tree species. While some food crops can form these associations, there has been limited research on how they might help overcome soil compaction. Some of the traits and mechanisms discussed in this systematic review that enable deep rooting in tree species can also assist food crops in dealing with soil compaction stress. Examination of these traits may help select food crops that could grow deeper into the soil parent material, particularly where there are degraded shallow soils and leptosols. A focus on these traits may capacitate us to select crop varieties and allow plant breeders to produce cultivars better able to penetrate and exploit soil parent materials to produce sustainable yields in profiles with shallow soils. However, further research is needed to explore how effectively food crops can grow in saprolite and the potential trade-offs regarding biomass allocation. Additionally, research should examine the disadvantages of promoting increased rooting in soil parent materials concerning geological processes, such as enhanced weathering, soil hydrology, and slope stability or instability.

## Supplementary Information

Below is the link to the electronic supplementary material.

Supplementary file1 (XLSX 457 KB)

Supplementary file2 (XLSX 910 KB)

## Data Availability

All relevant data are included in the article and its supporting materials. Data will be available upon request from the corresponding author.
